# The Utility of the Neutrophil-Lymphocyte Ratio as an Early Diagnostic Marker in Neonatal Sepsis

**DOI:** 10.7759/cureus.12891

**Published:** 2021-01-24

**Authors:** Santosh K Panda, Manas K Nayak, Soumini Rath, Palash Das

**Affiliations:** 1 Pediatric Medicine, Kalinga Institute of Medical Sciences, Bhubaneshwar, IND; 2 Pediatrics, Kalinga Institute of Medical Sciences, Bhubaneshwar, IND

**Keywords:** blood cell count, neutrophil lymphocyte ratio, c-reactive protein, infants, neonates

## Abstract

Aim

To find the diagnostic utility of the neutrophil to lymphocyte ratio (NLR) in the early diagnosis of neonatal sepsis.

Methodology

The case records of all blood culture-positive septic neonates admitted from January 2018 to December 2018 were reviewed. Total leucocyte count, absolute neutrophil count, absolute lymphocyte counts, NLR, and C-reactive protein (CRP) of septic neonates were compared with gestational age-matched nonseptic neonates by an unpaired t-test. The diagnostic performance of NLR and CRP was analyzed by receiver operating characteristic (ROC) analysis.

Result

A total of 41 blood culture-positive neonates and 52 nonseptic neonates were enrolled in this study. There was no significant difference in the total leucocyte count and absolute neutrophil counts of septic and nonseptic neonates. The mean absolute lymphocyte count of septic neonates (2795±1424/cumm) was significantly lower than that of nonseptic neonates (4449±1794/cumm; p=<0.001). The mean NLR of septic neonates (3.88±1.78) was significantly higher as compared to nonseptic (2.3404 ±1.98) neonates (p=0.045). For the diagnosis of sepsis, NLR at cutoff >1.7 had a sensitivity and specificity of 68.3% and 46.2%, respectively; CRP at cutoff >6 mg/dl had sensitivity and specificity of 78.05% and 92.31%, respectively. In the ROC analysis, the area under the curve (AUC) for CRP and NLR for the diagnosis of neonatal sepsis was 0.918 (p=<0.001) and 0.623 (p=0.042), respectively.

Conclusion

Blood culture-positive septic neonates had significantly higher NLR as compared to nonseptic neonates. However, when compared to CRP, NLR was not found to be a better predictor of sepsis in our study.

## Introduction

Sepsis refers to the clinical syndrome of systemic inflammation due to infection. Neonates are a particularly vulnerable population and neonatal sepsis is the second most common cause of mortality in this group [1]. Apart from mortality, it is associated with significant morbidity and long-term sequelae. Correct diagnosis and prompt initiation of therapy is, therefore, lifesaving, and overdiagnosis comes with its risks of inadvertent antibiotic exposure. Due to the poor yield of blood culture, various other markers are being tested, out of which C-reactive protein (CRP), procalcitonin, total leucocyte count, absolute neutrophil count, and the immature to total neutrophil ratio (I/T) have stood the test of time and have a good predictive value, especially when used together [2-3]. Complete blood count (CBC) is a part of the sepsis screen and is readily available in most places. Recently, the ratios of various components of CBC are being studied and applied in various clinical conditions in both pediatrics and adult studies [4-5]. The present study aims to assess the diagnostic utility of the neutrophil to lymphocyte ratio (NLR) in the early diagnosis of neonatal sepsis.

## Materials and methods

This retrospective study was conducted in the department of pediatrics, neonatology unit. It is a tertiary care neonatology unit having 40 beds, attached to a medical college, and caters to patients born in-house. It is also a referral unit to nearby districts and has approximately 1100 unique admissions for various neonatal conditions every year.

This study was conducted between January 2019 and January 2020. Records of all neonates having a positive blood culture were retrieved. Medical records and the complete blood count sent on the day of drawing blood culture were recorded. As per treatment protocol, for neonates presented with clinical symptoms and signs suggestive of neonatal sepsis, intravenous (IV) antibiotics were initiated after sending complete blood count (CBC), CRP, and blood culture. For blood culture, 0.5-1 ml blood was collected from venipuncture sites after disinfection of the site.

Various parameters, such as hemoglobin, packed cell volume, red cell distribution width, total white blood cell count, differential count, total platelet count, and mean platelet volume, of CBC were obtained from a Coulter counter, which was done using an automated analyzer (Beckman Coulter, LH 780, Brea, California). CRP was done using the latex-enhanced turbidometric immunoassay method with a photometer 5010 V5+ device (Agappe Diagnostic Ltd, Kerala, India). Blood culture-positive sepsis was diagnosed by the Bac T/AIert (bioMérieux, Marcy-l'Étoile, France) and VITEK-2 (bioMérieux) blood culture method, and the organism causing neonatal sepsis was recorded.

A similar number of gestational age-matched neonates, who had no clinical features suggestive of neonatal sepsis were designated as the control group. None of the control group neonates had received antibiotics, and all were discharged in healthy condition. Their complete blood cell parameters, CRP, and other relevant biochemical parameters were noted.

Statistical analysis

Continuous variables were statistically described in terms of mean and standard deviation (SD) and categorical variables as frequencies (number of cases) and percentages (%). A comparison of mean±SD of total leucocyte count, absolute neutrophil and lymphocyte counts, NLR, and CRP between the sepsis and control groups was done by an independent t-test. The diagnostic accuracy of NLR and CRP for neonatal sepsis was assessed by ROC curves and sensitivity, specificity, positive predictive value (PPV), and negative predictive value (NPV) were calculated. P-values <0.05 were considered statistically significant. All data were analyzed using the statistical software STATA 15.1 (StataCorp, College Station, Texas).

## Results

A total of 41 blood culture-positive septic neonates and 52 nonseptic neonates were enrolled for this study. The demographic profile of both groups of neonates is shown in Table 1.

**Table 1:** Demographic profile of septic and control neonates LSCS: lower segment cesarian section

Demographic variables	Sepsis(n=41)	Nonseptic(n=52)	P-value
Gestational age (weeks)	34.19 ± 4.70	33.78 ± 3.8	0.642
Birth weight (grams)	1980 ± 940	1920 ± 680	0.721
Mode of delivery (LSCS)	18	22	0.878
Sex (Male)	27	25	0.086

Amongst all the cases in the septic group, 16 babies were term gestation, seven babies were late preterm, and 18 babies were less than 34 weeks. Both the groups were comparable for weight and gestational age. There was no significant difference in the gender distribution of the patient or the mode of delivery in the septic and nonseptic groups. Among 41 blood culture-positive sepsis, 26 (63.41%) cases were gram-negative sepsis, 12 (29.26%) were gram-positive sepsis and three (7%) cases were fungal sepsis.

Among 41 culture-positive sepsis, 14 were early-onset sepsis and the rest were late-onset sepsis. Gram-negative sepsis was predominant over gram-positive and fungal sepsis in the septic group. The total leucocyte and their differential count, CRP of septic neonates, and nonseptic neonates are compared in Table 2.

**Table 2:** Comparison of hematological parameters of CBC and CRP between sepsis and nonseptic neonates ANC - Absolute neutrophil count, ALC - Absolute lymphocyte count, NLR - Neutrophil lymphocyte ratio, CRP - C-reactive protein, TLC - Total leucocyte count, CBC - Complete blood count

Hematological parameters	Sepsis(n=41)	Nonseptic(n=52)	P-value
TLC	12230±6752	14467±5496	0.082
ANC	8229± 5456	8366 ± 5462	0.905
ALC	2795 ±1424	4449 ±1794	<0.001
NLR	3.88 ± 1.78	2.3404 ±1.98	0.045
CRP (mg/dl)	43.50 ± 26.76	3.39 ± 1.50	<0.001

**Figure 1:**
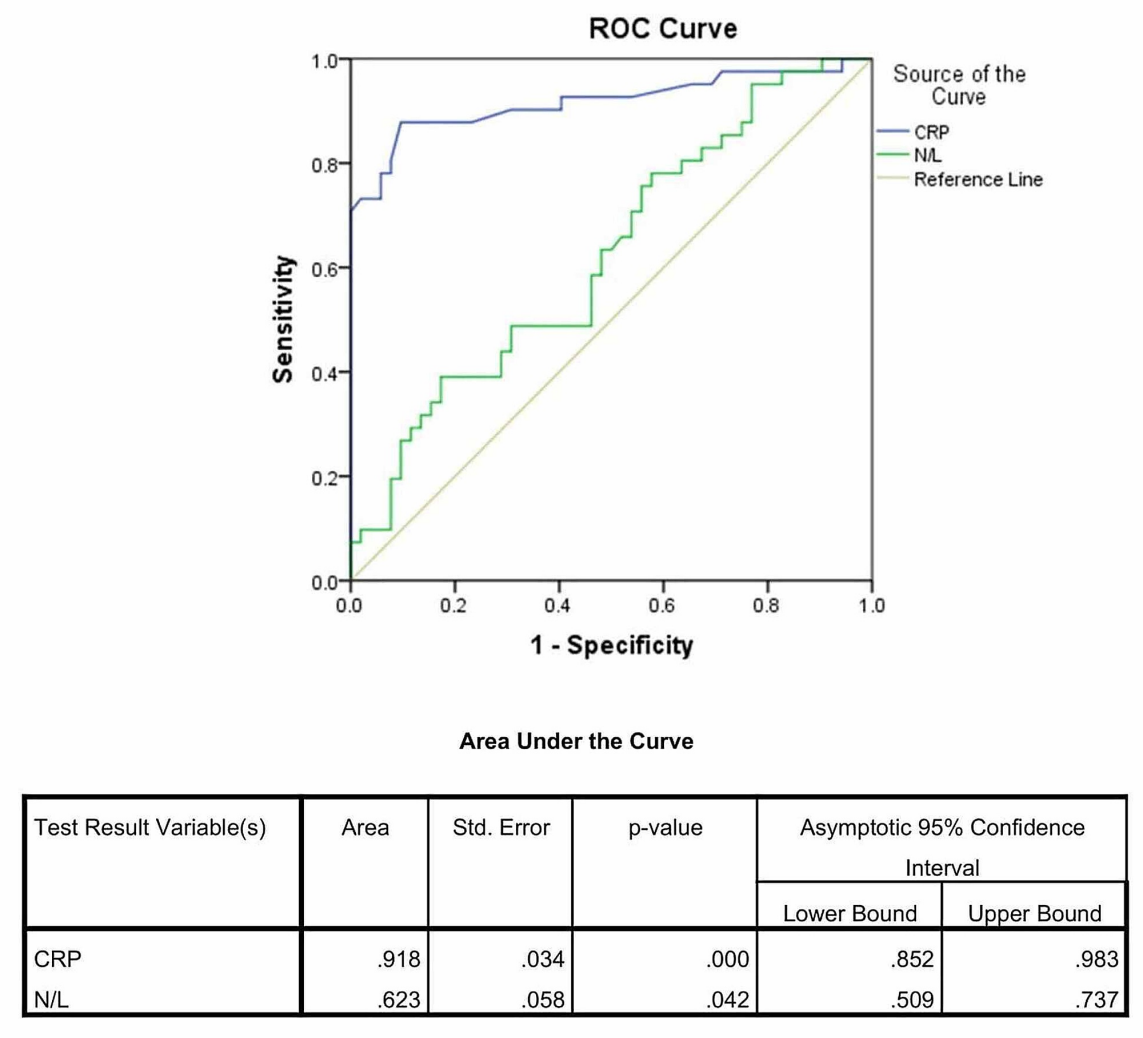
Receiver operating curve of CRP and NLR in neonatal sepsis CRP - C-reactive protein, NLR - Neutrophil lymphocyte ratio

Five out of 41 blood culture-positive septic neonates had leucopenia (total leucocyte count (TLC)<5000/mm^3^) and none of the neonates in the nonseptic group had leucopenia. There was no significant difference in total leucocyte count and absolute neutrophil count of septic and nonseptic neonates. The mean absolute lymphocyte counts of septic neonates (2795±1424) were significantly (p=<0.001) lower than nonseptic neonates (4449±1794). The NLR of septic neonates (3.88±1.78) was significantly higher as compared to nonseptic (2.3404±1.98) neonates (p=0.045). The NLR at cutoff > 1.7 had a sensitivity of 68.3% and specificity of 46.2% for the diagnosis of sepsis. Table 3 shows the sensitivity, specificity, PPV, and NPV of both NLR and CRP for neonatal sepsis. In the ROC analysis, the AUC for CRP and NLR for the diagnosis of neonatal sepsis were 0.918 (p=<0.001) and 0.623 (p=0.042), respectively (Figure 1).

**Table 3:** The sensitivity, specificity, positive predictive value (PPV), negative predictive value (NPV) of the CRP and N/L ratio CRP - C-reactive protein, N/L - neutrophil/lymphocyte

Diagnostic test	Cut-off point	Sensitivity	Specificity	PPV	NPV
CRP	(>=6 mg/dl)	78.05%	92.31%	88.88%	84.21%
N/L	(>1.7)	68.3%	46.2%	50%	64.86%

## Discussion

In context to previous studies, the inclusion of NLR as a newer diagnostic marker in the early diagnosis of neonatal sepsis was supported in this study. In neonatal care, diagnostic tests are utilized in asymptomatic neonates with high risk for early-onset sepsis and symptomatic babies to confirm the presence or absence of infective condition based on sensitivity, specificity, positive predictive value, and negative predictive values in the early stages of the disease [6].

The sensitivity of NLR (68%) was similar to CRP (78%) for the diagnosis of neonatal sepsis but NLR (46%) had lower specificity as compared to CRP (92%) in this study. Dynamic changes in neutrophils and lymphocytes occur during neonatal sepsis. Based on bone marrow production, they are deployed during infection to the tissue site. Apoptosis of the involved cell line is also affected. During sepsis, the lifespan of neutrophils increases due to decreased apoptosis process mediated by a decreased caspase3 level and NF-kB activation [7-8].

Neutrophils act as the first-line defense after the invasion of microbes. They function through phagocytosis and are modulated at various steps by multiple factors. The body controls immune-mediated tissue damage by lymphocyte by apoptosis. Due to the migration of lymphocytes to the site of infection, lymphocytopenia is also seen in sepsis [9-10].

The majority of septic neonates had a normal total leukocyte count, so the presence of a normal white blood cell (WBC) count cannot rule out sepsis. This finding is incongruous to Sucilathangam et al., where they found that 85% of culture-positive neonates had a normal WBC count [11]. Similarly, Sumitro et al. reported in their study that around 90% of septic neonates had a WBC count within the normal range [12]. In two multicentric studies, the WBC count and ANC count were studied, and they did not perform well as a standalone diagnostic tool for early-onset neonatal sepsis in late-preterm and term neonates. The extreme value of WBC <1000/µL and ANC <100/µL had a high likelihood ratio for EOS but with very low sensitivity [13-14].

As per some authors, with a decreasing incidence of EOS in developed countries, the utility of WBC count in the early neonatal period is trending down [15]. Clinicians used to follow the Manroe and Mouzinho postnatal age nomogram for total leucocyte count using specified cutoffs after >34 weeks of gestation and for preterm neonates, respectively [16-17]. The normal range of leukocyte count varies with gestational age, mode of delivery, site of sampling, and altitude while many perinatal conditions like maternal pregnancy-induced hypertension, birth asphyxia, and hemolytic disease influence neutrophil count [18-19].

A higher NLR in septic groups as compared to healthy neonates was previously reported in both the preterm and term populations. In a study by Omran et al., NLR values of septic term neonates (2.9 ± 1.7) were higher compared to nonseptic term neonates (1.6 ± 0.4) and NLR at a cutoff of 2.7 diagnosed sepsis with sensitivity 80% and 57.1% specificity [20]. Can et al. found the neutrophil count and NLR in term onset septic groups were higher as compared to healthy neonates. The mean ± SD of NLR in term sepsis and nonseptic neonates was 2.88 ± 0.16 and 0.21 ± 0.12, respectively. NLR at a cutoff value of 1.76 diagnosed term neonatal early-onset sepsis with sensitivity 97.4% and specificity 100% and had a good diagnostic yield with AUC =0.99 in ROC analysis [21].

Ozdemir et al. studied and found preterm late-onset sepsis neonates had a higher mean NLR (3.69 ± 3.0) as compared to cultures negative babies (1.56 ± 1.83) and NLR at cutoff 1.7 had a sensitivity of 73.1% and specificity of 78.7% for length of stay (LOS) [22]. In adults, severe sepsis and septic shock NLR was measured at admission and was seen as a significant predictor of mortality [23].

In this study, the NLR of septic neonates was 3.88 ±1.78 and was significantly higher as compared to nonseptic neonates, which was 2.34±1.98. This study was different from the above studies since here the neonatal population had enrolled term, preterm, early-onset, and late-onset sepsis neonates. Babies who presented within the first 72 hours of birth with signs of sepsis were diagnosed as early-onset sepsis while those who presented later were classified as late-onset sepsis. In a similar kind of study population from Indonesia, the median NLR value of septic neonates 3.63 was higher compared to an NLR of 2.12 in the suspected sepsis group, with an AUC of 0.63 [12].

CRP is an acute-phase reactant. It belongs to the pentraxin family and is produced in the liver and rises after 10-12 hours of infection. CRP has been proved over the years to have good sensitivity and specificity for the diagnosis of neonatal sepsis. Various studies have reported the sensitivity and specificity of CRP in the range of 35%-94% and 60%-96%, respectively [24]. In this study, the AUC for CRP and NLR was statistically significant for the diagnosis of sepsis. The AUC of CRP was higher compared to NLR AUC, suggesting a better diagnostic yield of CRP over NLR. In a study by Hamiel et al., better diagnostic validity was obtained by combining CRP and NLR in comparison to CRP or NLR alone too [25].

## Conclusions

The study was a monocentric retrospective study with relatively small sample size. The maternal characteristics, neonatal clinical characteristics, and timing of neonatal sepsis were not stratified. The strength of the study was that all septic neonates were blood culture-positive sepsis and all non-septic babies were discharged from the hospital in stable condition without receiving any antibiotics. Hence, the diagnostic yield of NLR for neonatal sepsis could be researched further in a prospective multicentric study separately in early and late-onset neonatal sepsis.
